# Hydrodynamic conditions affect the proteomic profile of marine biofilms formed by filamentous cyanobacterium

**DOI:** 10.1038/s41522-022-00340-w

**Published:** 2022-10-17

**Authors:** Maria J. Romeu, Dany Domínguez-Pérez, Daniela Almeida, João Morais, Mário J. Araújo, Hugo Osório, Alexandre Campos, Vítor Vasconcelos, Filipe J. Mergulhão

**Affiliations:** 1 LEPABE – Department of Chemical Engineering, Faculty of Engineering, University of Porto, Rua Dr. Roberto Frias s/n, 4200-465 Porto, Portugal; 2 ALiCE - Associate Laboratory in Chemical Engineering, Faculty of Engineering, University of Porto, Rua Dr. Roberto Frias s/n, 4200-465 Porto, Portugal; 3 CIIMAR – Interdisciplinary Centre of Marine and Environmental Research, University of Porto, Terminal de Cruzeiros do Porto de Leixões, Av. General Norton de Matos s/n, 4450-208 Matosinhos, Portugal; 4 Department of Biological Sciences, Clemson University, 055A Life Science Facility, 190 Collings Street, Clemson, SC 29634 US; 5 i3S – Instituto de Investigação e Inovação em Saúde, Universidade do Porto, 4200-135 Porto, Portugal; 6 Institute of Molecular Pathology and Immunology of the University of Porto, IPATIMUP, Rua Júlio Amaral de Carvalho 45, 4200-135 Porto, Portugal; 7 Faculty of Medicine, University of Porto, Al. Prof. Hernâni Monteiro, 4200-319 Porto, Portugal; 8 Department of Biology, Faculty of Sciences, University of Porto, Rua do Campo Alegre, 4169-007 Porto, Portugal

**Keywords:** Biofilms, Cellular microbiology

## Abstract

Proteomic studies on cyanobacterial biofilms can be an effective approach to unravel metabolic pathways involved in biofilm formation and, consequently, obtain more efficient biofouling control strategies. Biofilm development by the filamentous cyanobacterium *Toxifilum* sp. LEGE 06021 was evaluated on different surfaces, glass and perspex, and at two significant shear rates for marine environments (4 s^−1^ and 40 s^−1^). Higher biofilm development was observed at 4 s^−1^. Overall, about 1877 proteins were identified, and differences in proteome were more noticeable between hydrodynamic conditions than those found between surfaces. Twenty Differentially Expressed Proteins (DEPs) were found between 4 s^−1^ vs. 40 s^−1^. On glass, some of these DEPs include phage tail proteins, a carotenoid protein, cyanophynase glutathione-dependent formaldehyde dehydrogenase, and the MoaD/ThiS family protein, while on perspex, DEPs include transketolase, dihydroxy-acid dehydratase, iron ABC transporter substrate-binding protein and protein NusG. This study contributes to developing a standardized protocol for proteomic analysis of filamentous cyanobacterial biofilms. This kind of proteomic analysis can also be useful for different research fields, given the broad spectrum of promising secondary metabolites and added-value compounds produced by cyanobacteria, as well as for the development of new antibiofilm strategies.

## Introduction

Over the past years, eutrophication and climate changes have been promoting cyanobacterial blooms in aquatic environments^1^. In addition to threatening the ecosystem, this phenomenon also degrades water quality for fisheries and public health^2^. Cyanobacteria can also affect animal and human health through their ability to produce cyanotoxins^3–6^. These toxins can also have detrimental effects through ingestion of contaminated food or even by drinking water during recreational activities^7^. Moreover, cyanobacteria blooms from benthic mat proliferations have increased worldwide^1^. Indeed, cyanobacteria are the major constituents of marine biofilms, and they respond quickly to environmental pattern changes, which allows them to have a diverse distribution. Besides the contamination of aquaculture facilities^8^, marine biofilms cause problems related to the corrosion of different facilities and submerged equipment^9^. Marine biofouling can also lead to increased fuel consumption in ships and associated pollution^10,11^, as well as the spread of invasive non-indigenous species on marine vessels between different ecosystems^12^. Overall, marine biofouling challenges include environmental, as well as economic and health concerns^13^.

Several cues have been identified regarding cyanobacterial adhesion and biofilm formation, particularly for *Synechocystis*^14–17^*, Thermosynechococcus elongatus*^18^, *Microcystis aeruginosa*^19^, *Anabaena* sp. PCC 7120^20^, *Synechococcus elongatus* PCC 7942^21^ and *Nostoc punctiforme*^15^.

Advancements in omics technologies are continuously progressing. Although cyanobacteria proteomics has dealt with many technical challenges^22^, cyanobacteria molecular biology is a promising field for further advancements^23,24^. The fouling surface properties and shear rate are two parameters that have a critical impact on marine biofilms^25,26^. In a previous qualitative proteomic study, the proteomic profile of biofilm cells from two cyanobacterial strains developed on glass and perspex, and at different shear forces was analysed^27^. The results revealed that variances in protein composition were more noticeable in biofilms formed under different hydrodynamic conditions than in those formed on different surfaces^27^. However, to identify pathways that are triggered under different circumstances to which cells were subjected, quantitative proteomic analyses are the most relevant tool^28,29^. A recent quantitative study performed with the unidentified filamentous cyanobacterium LEGE 06007 revealed 41 Differentially Expressed Proteins (DEPs)^30^. The proteomic profile can help in the characterization of virulence factors and cellular processes involved in biofilm regulation. Besides improving the fundamental understanding of biofilm development and behavior, the identification of these proteins using high throughput proteomic tools provides new insights into the response to stress conditions, including cell signaling and stress response pathways that are activated under different conditions. Therefore, as more proteins and their roles in biofilm development are explored, novel potential targets for biofilm control may be discovered. Moreover, by knowing which proteins are produced under stress conditions and which metabolic pathways are activated, it may be possible that new metabolites can be identified with antibiofilm properties. Cyanobacteria are known to produce nearly 800 bioactive secondary metabolites, and it is known that cyanobacteria are able to interfere with other organisms in their communities through the release of compounds into the surrounding medium^31^. The isolation of these metabolites led to the discovery of a strong inhibitory activity against other marine organisms and is currently being explored for the production of antifouling coatings^32^. However, data available on cyanobacterial proteomic are still scarce^33^ and most of the studies focus on well-studied model cyanobacteria, such as the unicellular cyanobacterium *Synechocystis* sp. PCC 6803^34^. Different cyanobacterial strains should be considered, and this is of particular importance for filamentous cyanobacterial strains, in which the stress and predation resistance, as well as the improved resource acquisition, may be beneficial^35^. However, the standardization of the proteomic protocol in these organisms is a hard task, and the knowledge needs to be increased incrementally. This work mainly focuses on the improvement of proteomic methodology as well as on the significance of the results obtained and the raw data availability. Due to the high production of cellulose-rich pigments by cyanobacteria, all sample preparation, protein extraction and accurate quantification is a difficult endeavor and hinder the standardization of the proteomic protocol for these organisms. This study contributes to the development of a standardized protocol for proteomic analysis of filamentous cyanobacterial biofilms, enabling other groups to advance their research in this field and facilitating inter-lab comparisons. Moreover, this kind of proteomic analyses can also be useful for different research fields given the broad spectrum of promising secondary metabolites and added-value compounds produced by cyanobacteria^36^ that can be used in biotechnological applications, cosmetic, feed and food industries^37^ and medical purposes^38^. Synechococcales order includes both filamentous and single-celled types and involves several cyanobacteria genera, among which the ubiquitous *Nodosilinea, Synechococcus, Cyanobium* and *Leptolyngbya* genus. Unidentified Synechococcales include about 32 different strains, and some of them are able to produce common cyanotoxins such as β-methylamino-L-alanine^39,40^. These strains can be found in different ecosystems like brackish water, freshwater, marine, and even hypersaline environments. Moreover, cyanobacterial strains from Synechococcales are present in distinct geographies around the world, including Portugal, Chile, Mexico, and Morocco^39^. The Blue Biotechnology and Ecotoxicology Culture Collection (LEGE-CC) is a biological resource center, which among several additional organisms, maintains different cyanobacterial strains^39,40^. The unidentified filamentous Synechococcales LEGE 06021 strain was used in this study, given the relevance of cyanobacteria on marine biofouling. To complete the current study, a genus identification of this cyanobacterial strain was performed. This study addresses biofilm development of this filamentous cyanobacterial strain, as well as its proteomic profile when subjected to different conditions of shear and surfaces. Therefore, this study aims to characterise the biofouling potential of this filamentous cyanobacteria, on a long-term assay, under two different hydrodynamic conditions and with two surfaces, as well as to explore the quantitative proteomic profile under these different biofilm development conditions.

## Results

### Phylogenetic analysis

**Fig. 1:**
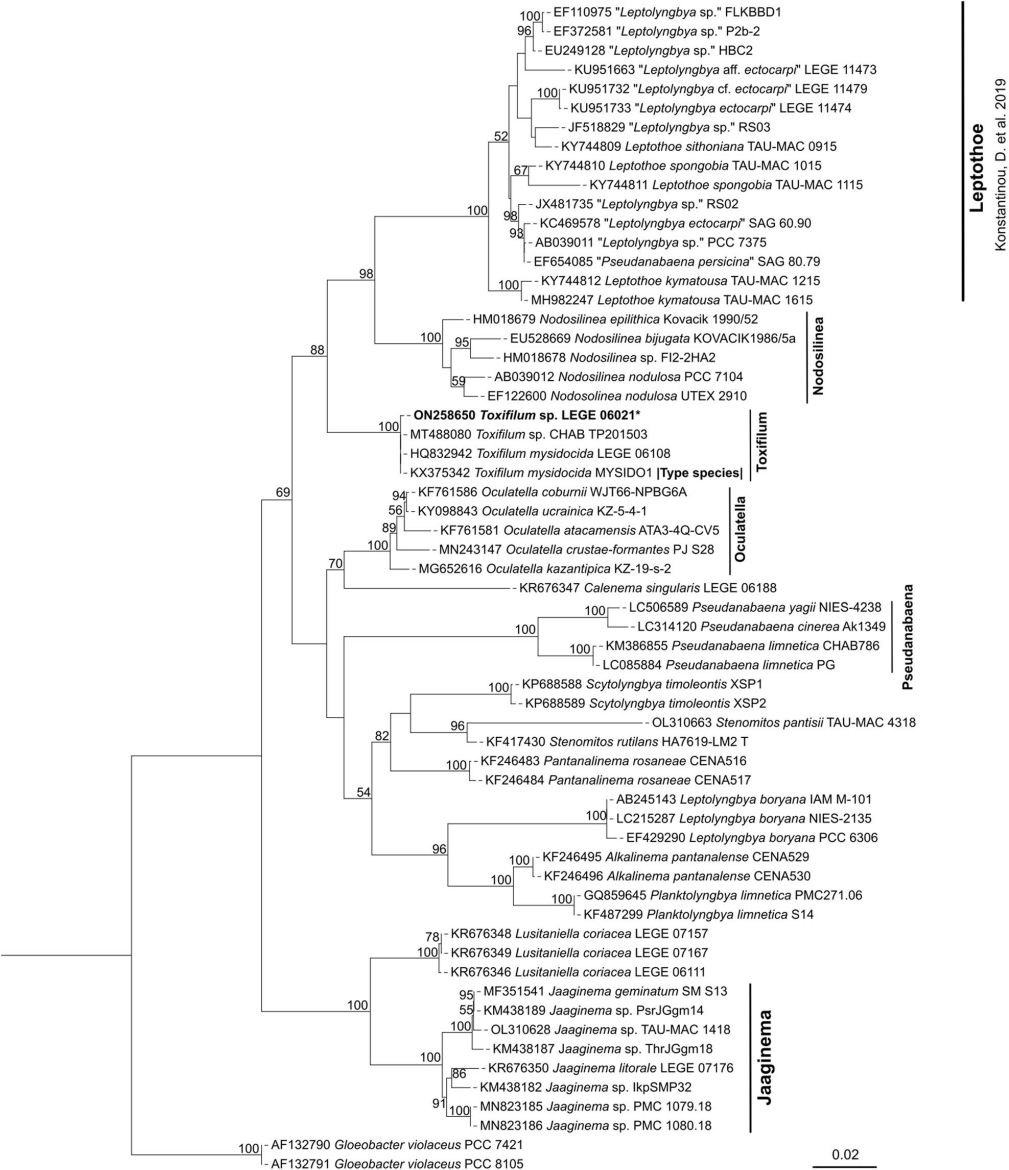
Maximum likelihood (ML) phylogenetic tree based on 61 partial 16S rRNA gene sequences of cyanobacteria strains belonging to the orders Pseudanabaenales and Synechococcales. *Gloeobacter violaceus* PCC 7421 and *Gloeobacter violaceus* PCC 8105 were used as outgroup. Phylogenetic position of *Toxifilum* sp. LEGE 06021 is indicated in bold. Bootstrap values over 50% are indicated at the nodes. Black stars represent the strains whose sequences were obtained in this work. Strains labeled with quotes indicate that names correspond to Genbank labels.

Phylogenetic relationships of unidentified filamentous Synechococcales LEGE 06021 and other relatives were investigated by using a maximum likelihood phylogenetic tree constructed from their partial 16S rRNA gene sequences (Fig. 1). Our phylogenetic results confirmed that this strain belongs to the *Toxifilum* genus (related to the type species *Toxifilum mysidocida)*^41^.

### Biofilm development analysis

**Fig. 2:**
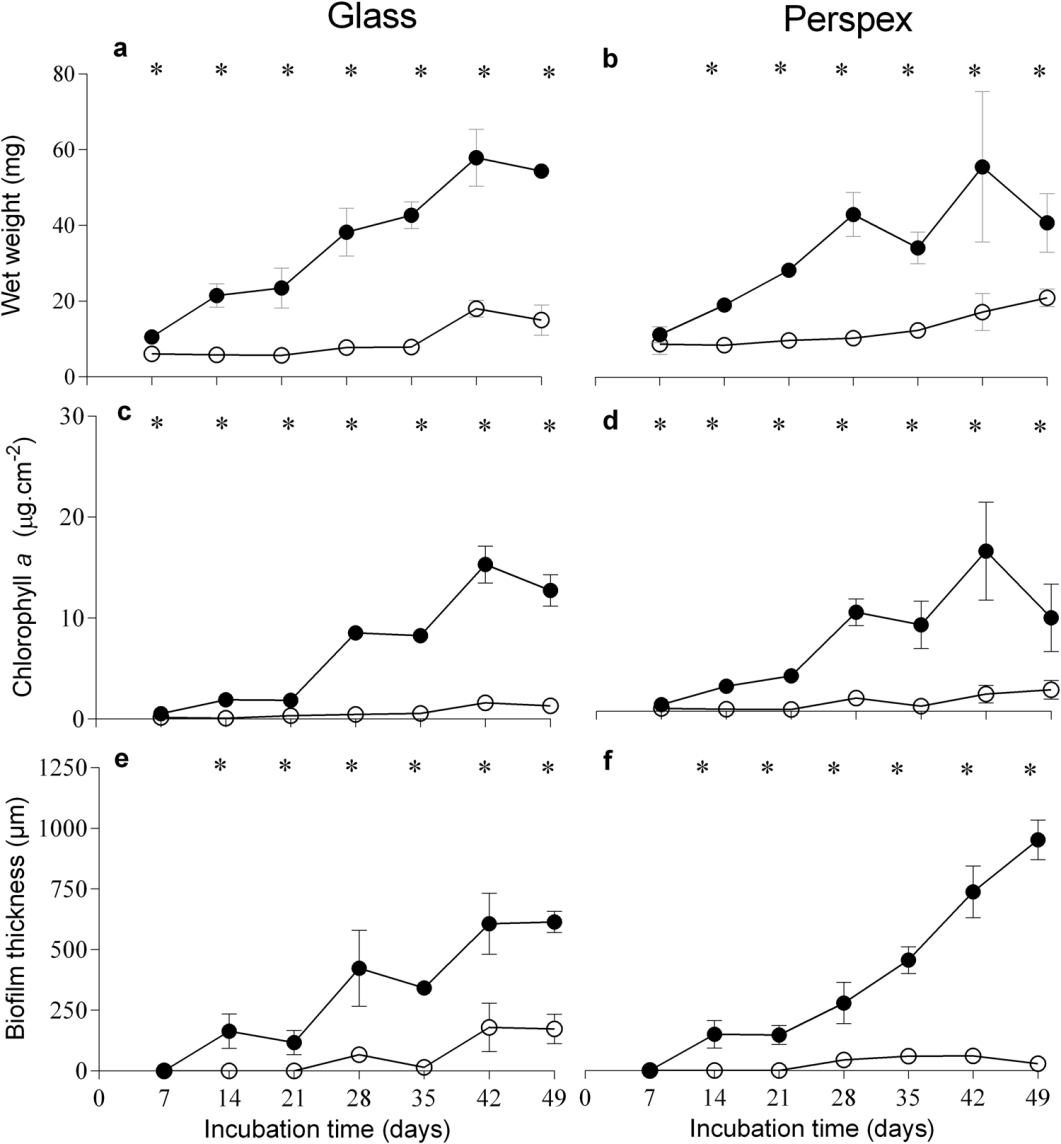
Evaluation of *Toxifilum* sp. LEGE 06021 biofilm development. The parameters analysed refer to wet weight (**a**, **b**), chlorophyll *a* quantification (**c**, **d**) and biofilm thickness (**e**, **f**). Biofilms were formed in glass (**a**, **c**, **e**) or perspex (**b**, **d**, **f**), at two average shear rates, 4 s^−1^ (closed circles) and 40 s^−1^ (open circles) for 49 days. Standard deviations from two biological assays with three replicates each are represented. Symbol * indicate statistically different values for *P* < 0.05 (unpaired *t*-tests) at each incubation time.

**Fig. 3:**
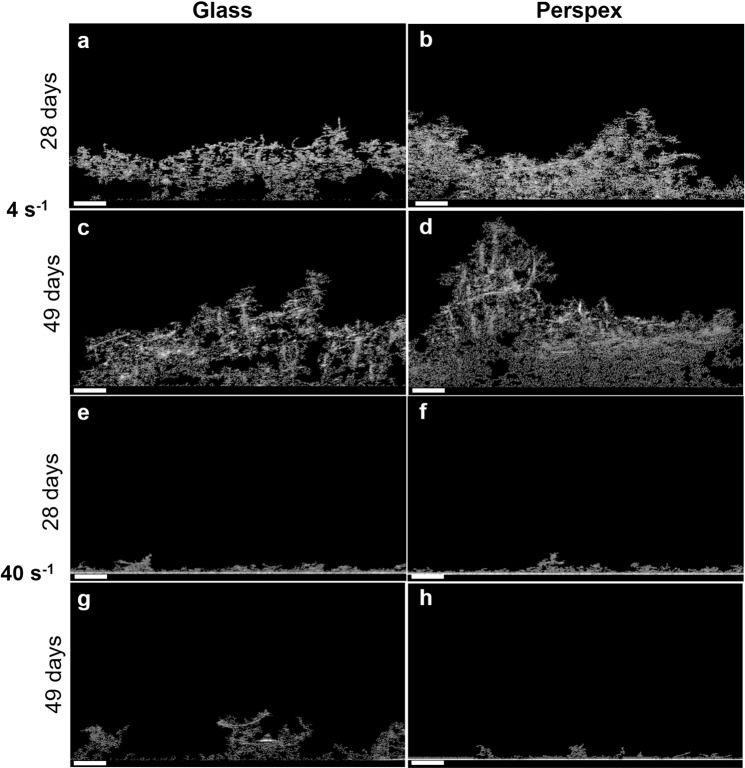
2D cross-sectional OCT images of *Toxifilum* sp. LEGE 06021 biofilms. The representative images show biofilms formed on glass and perspex at two different shear rates, 4 s^−1^ (**a**–**d**) and 40 s^−1^ (**e**, **f**), and the biofilm structure evolution from days 28 to 49. Scale bar = 200 μm.

Wet weight, chlorophyll *a* content and biofilm thickness evolution from cyanobacterial biofilm under different conditions were assessed for 49 days (Fig. 2). Biofilm biomass was higher at the lowest shear rate on both surfaces. However, this tendency was more evident for biofilm thickness values obtained from perspex (Fig. 2f). Biofilm mass obtained on the glass surface under lower shear conditions (4 s^−1^) was on average 70.5% higher (Fig. 2a) when compared to the values obtained at a higher shear rate (40 s^−1^), whereas on perspex, biofilm wet weight was on average 57.3% higher (Fig. 2b). The chlorophyll *a* content at 4 s^−1^ on glass was on average 87.5% higher (Fig. 2c) than at 40 s^−1^, on perspex, it was, on average 83.9% higher (Fig. 2d). Regarding biofilm thickness, values obtained on glass under lower shear conditions were on average 87.0% higher (Fig. 2e), whereas on perspex, they were on average 93.3% higher (Fig. 2f). Moreover, the highest value of biofilm thickness was observed at 4 s^−1^ on perspex (around 955.9 μm; Fig. 2f, 49 days). When biofilms developed under different surfaces at the same hydrodynamic conditions were compared, similar values from these four parameters were obtained (Supplementary Fig. 1). Figure 3 shows representative 2D cross-sectional images obtained by Optical Coherence Tomography (OCT) at 28 days and at the end of the experiment (49 days). This period corresponds to a maturation phase of biofilm development and the time at which major differences between the two hydrodynamics conditions were observed (Fig. 2).

### Proteomic analysis

At the last sampling time, biofilm samples of *Toxifilum* LEGE 06021 were recovered for proteomic analysis. Altogether, 1877 proteins were identified from 24 biofilm samples of four different biofilm conditions using a shotgun proteomics approach (Supplementary Table 1). Among these proteins, 54.2% are related to metabolic processes, and 23.5% to proteomic metabolism (Supplementary Fig. 2).

**Fig. 4:**
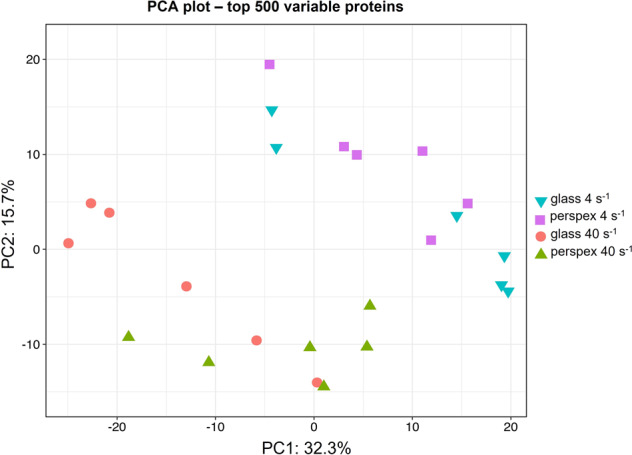
Principal Component Analysis (PCA) of the proteins identified from marine biofilms formed by filamentous cyanobacteria under four different biofilm growing conditions. The PCA plot displays a grouping pattern among the six replicates studied from each biofilm condition formed on glass and perspex, but some differences were observed between the two hydrodynamic conditions studied (4 s^−1^ and 40 s^−1^). The two principal components extracted (PC1 and PC2) can explain 48% of the total variability revealed among the proteins analysed. PCA was performed with the respective function provided within the “DEP” R package^101^, used for the downstream proteomic analyses.

**Fig. 5:**
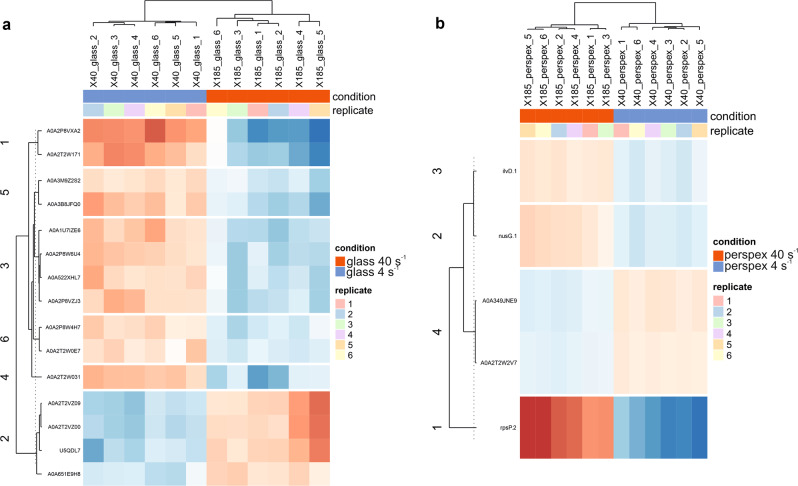
Heatmaps of the Differentially Expressed Proteins (DEPs) identified between comparisons of the same surface studied at different hydrodynamics conditions. The figures display the DEPs resulting from the comparisons between the conditions (**a**) 4 s^−1^ *vs*. 40 s^−1^ on glass clustered in six groups by k-means clustering, and (**b**) 4 s^−1^ *vs*. 40 s^−1^ on perspex clustered in four groups. The rows show the corresponding UniProt accession of DEPs with their corresponding expression in the columns as the log_2_ fold-change (red: enriched; blue: depleted) of the intensity from each replicate.

**Fig. 6:**
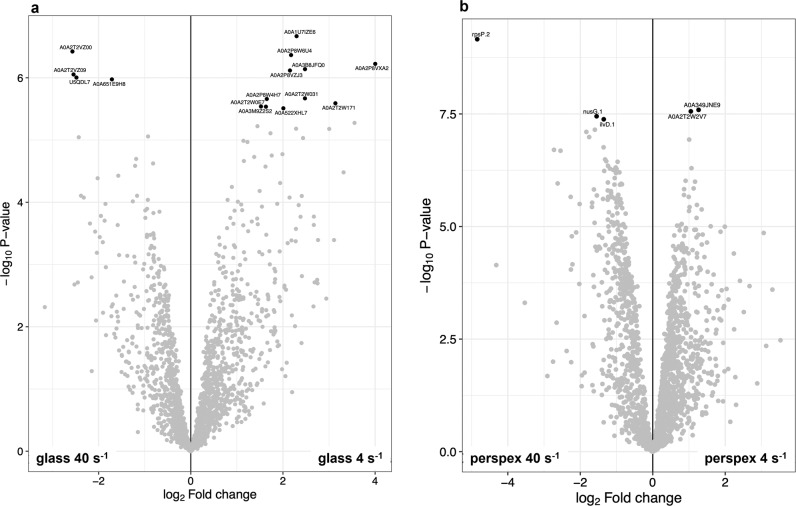
Volcano plot of the differentially expressed proteins (DEPs) identified between comparisons of the same surface studied at different hydrodynamics conditions. The figure displays the DEPs (black dots) and their corresponding accession/gene names, as the resulting *P* values (−log_10_) in the y-axis, plotted against the fold changes (log_2_) in the x-axis, obtained between the comparisons 4 s^−1^ *vs*. 40 s^−1^ on glass (**a**) and on perspex (**b**).

**Fig. 7:**
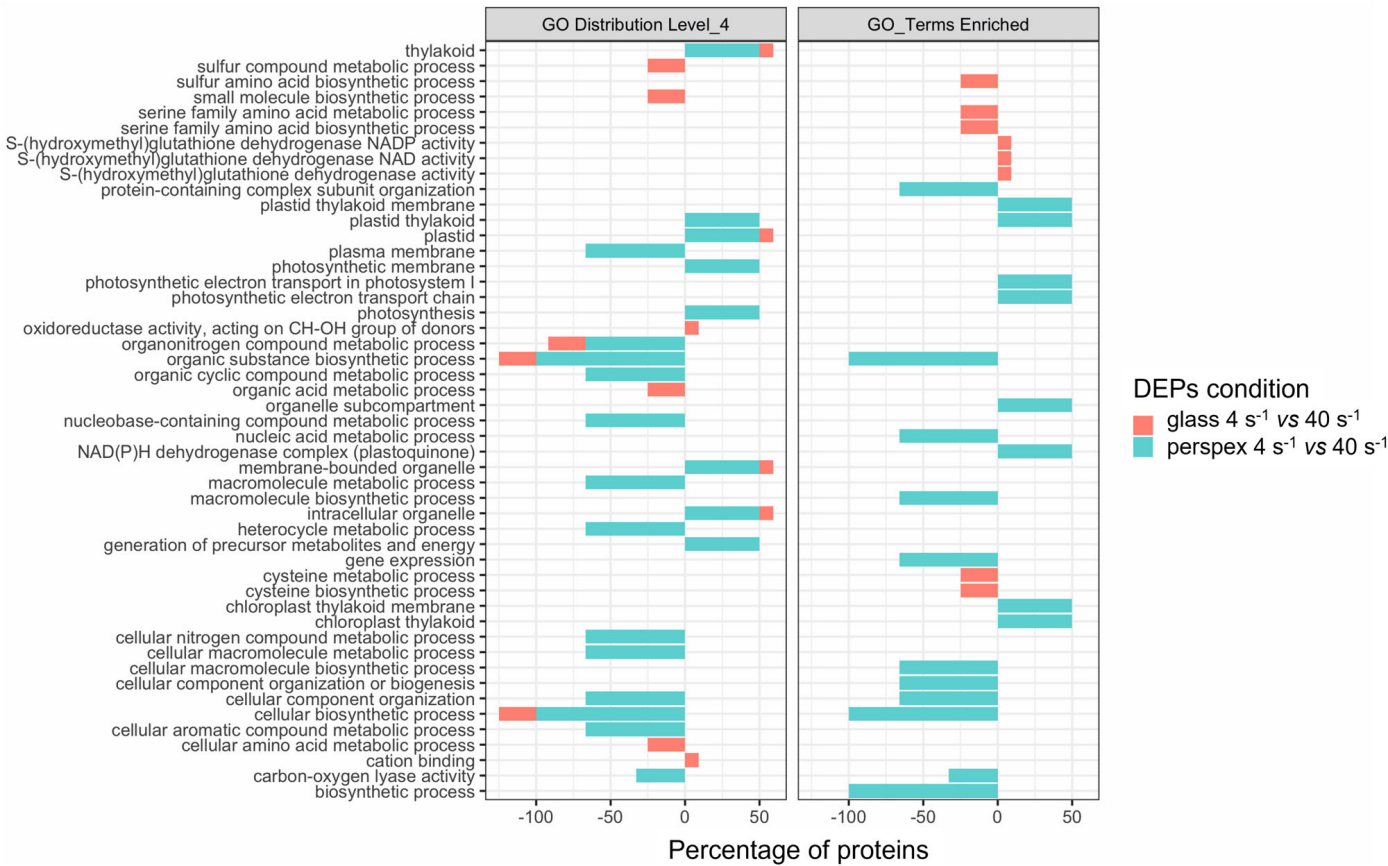
Gene Ontology (GO) distribution and GO enrichment of Differentially Expressed Proteins (DEPs). In the left panel is shown the GO distribution by level 4 of the DEPs, whereas the right panel displays the corresponding enriched GO terms. The divergent stacked bars (negative and positive) represent the percentage of DEPs (x-axis) matching each GO Category (y-axis), relative to the number of DEPs found at both sides (up/down) of the tested conditions (DEPs Conditions). The total number of DEPs obtained from each comparison were 11-up/4-down (from glass 4 s^−1^ vs. 40 s^−1^) and 2-up/3-down (from perspex 4 s^−1^ vs. 40 s^−1^). The GO annotation was performed with the OmicsBox software version 1.4.11, as well as the enrichment analyses (Fisher’s exact test, *P* < 0.05).

The Principal Component Analysis (PCA) plot revealed a similar pattern among replicates of the same conditions, whereas some differences were visualized between the different shear rates for the same surface used (Fig. 4). As revealed by the macroscopic evaluation of cyanobacterial biofilm, the proteomic analysis also showed a higher difference in protein expression between the two hydrodynamic conditions than those found between the two surfaces. Indeed, these differences between different hydrodynamic conditions were quantitatively significant, resulting in 20 DEPs, of which 15 DEPs were found between the comparison at 4 s^−1^ *vs*. 40 s^−1^ on glass (Figs. 5a and 6a), whereas five DEPs were found between samples from biofilms developed at 4 s^−1^ *vs*. 40 s^−1^ on perspex (Figs. 5b and 6b). Most of these DEPs (around 50%) were found as up-regulated at 4 s^−1^ *vs*. 40 s^−1^ on glass (Fig. 6a). Like the GOs Categories of the two up-regulated DEPs found in 4 s^−1^ *vs*. 40 s^−1^ on perspex, these 11 up-regulated DEPs found in 4 s^−1^ *vs*. 40 s^−1^ on glass are related to cellular components (intracellular organelle, plastid, thylakoid, membrane-bounded organelle), cation binding and oxidoreductase activity (Fig. 7).

From all of DEPs found on glass, a fragment of condensation protein, uncharacterized proteins, PRC domain-containing proteins, one orange carotenoid protein, and enzymes like cyanophynase and glutathione-dependent formaldehyde dehydrogenase were identified as up-regulated at 4 s^−1^ (Table 1). Among these uncharacterized proteins, two disordered proteins (A0A2T2W171 and A0A522XHL7), which are more likely to perform their function under extreme conditions^42^, were also identified. Glutathione-dependent formaldehyde dehydrogenase seems to have a strong impact on biofilm development at this condition since this subset of 11 DEPs contained three enriched GO terms related to S-(hydroxymethyl) glutathione dehydrogenase NAD/NADP activity (Fig. 7). Overall, all these up-regulated proteins found at 4 s^−1^ on glass are related to the photosynthetic process (Fig. 6). On the other hand, two phage tail proteins, a MoaD/ThiS family protein and an uncharacterized protein with a disordered domain, were found as down-regulated proteins on glass at 4 s^−1^ when compared with biofilm cells from 40 s^−1^ (Table 1).

**Table 1:** List of the DEPs found between comparisons of the same surface studied at different hydrodynamics conditions.

UniProt accession	Description	Glass 4 s^−1^ *vs*. 40 s^−1^ (LFC)	Glass 4 s^−1^ *vs*. 40 s^−1^ (p.adj)	Perspex 4 s^−1^ *vs*. 40 s^−1^ (LFC)	Perspex 4 s^−1^ *vs*. 40 s^−1^ (p.adj)
A0A1U7IZE6	Orange carotenoid protein	2.3	0.01		
A0A2P8VXA2	Glutathione-dependent formaldehyde dehydrogenase	4	0.01		
A0A2P8VZJ3	Uncharacterized protein	2.15	0.01		
A0A2P8W4H7	Uncharacterized protein	1.65	0.03		
A0A2P8W6U4	PRC domain-containing protein	2.18	0.01		
A0A2T2VZ00	Phage tail protein	−2.57	0.01		
A0A2T2VZ09	Phage tail protein	−2.55	0.01		
A0A2T2W031	PRC domain-containing protein	2.48	0.03		
A0A2T2W0E7	Uncharacterized protein	1.52	0.04		
A0A2T2W171	Uncharacterized protein	3.14	0.03		
A0A3B8JFQ0	Condensation protein (Fragment)	2.48	0.01		
A0A3M9Z2S2	Cyanophycinase	1.63	0.04		
A0A522XHL7	Uncharacterized protein	2.01	0.04		
A0A651E9H8	MoaD/ThiS family protein	−1.71	0.01		
U5QDL7	Uncharacterized protein	−2.48	0.01		
A0A2T2W2V7	Transketolase			1.05	0.04
A0A349JNE9	Iron ABC transporter substrate-binding protein			1.27	0.04
A0A6G3Z7U9^a^	Dihydroxy-acid dehydratase			−1.35	0.05
A0A651DR21^b^	Transcription termination/antitermination protein NusG			−1.55	0.05
A0A651DTD2^c^	30 S ribosomal protein S16			−4.84	0

The corresponding UniProt accession of DEPs is provided with their corresponding gene and a brief description, as well as the log 2-fold-changes (LFC) and the resulting and adjusted *P*-values (p.adj) for each comparison (4 s^−1^ *vs*. 40 s^−1^ on glass, and 4 s^−1^ *vs*. 40 s^−1^ on perspex).

Gene names: ^a^ilvD; ^b^nusG; ^c^rpsP.

On perspex, DEPs include iron ABC transporter substrate-binding protein and transketolase, as up-regulated proteins, and transcription termination/antitermination protein NusG, dihydroxy-acid dehydratase, and 30S ribosomal protein S16 as down-regulated proteins at 4 s^−1^ (Table 1). More details about DEPs description and their GO annotation can be found in Supplementary Table 2.

## Discussion

Chlorophyll *a* content monitorization proved to be a useful tool for following cyanobacterial biofilm growth since a correlation between chlorophyll *a* production and biofilm wet weight was observed (Supplementary Fig. 3), as already reported for different filamentous cyanobacterial strains^27,30,43^. In the present study, differences in biofilm development at different shear conditions were noticed in the early stages of biofilm development, but major differences were found in the maturation phase of biofilm development, as previously observed for filamentous^27,30,43^ and coccoid cyanobacteria^25^. Probably, the increased shear rate has a weaker impact during adhesion and initial biofilm formation, but later on, it may promote sloughing and erosion events, affecting biofilm cohesion and structure. At lower fluid velocities, external mass transfer of nutrients to the top layers of the biofilm is reduced and this may reduce diffusion to the inner layers of the biofilm^44^. It is therefore possible that the biofilm adapts its architecture to facilitate this nutrient transfer as previously shown for other bacterial biofilms produced under flow conditions^45^. Moreover, the lower biofilm biomass obtained under a higher shear rate (Fig. 3e–h) does not allow a detailed analysis of the biofilm structure. However, given the results obtained from biofilm thickness (Fig. 2e, f), in which the effect of fluid velocity was more noticeable during biofilm maturation rather than during cell adhesion, it seems that the increased shear stress was a significant obstacle to biofilm development at 40 s^−1^ most likely due to erosion/sloughing effects. Despite the different properties of glass and perspex, which is the most hydrophobic surface^43^, the finding that the effect of surface hydrophobicity was less important than the hydrodynamic effect on cyanobacteria biofilm development had already been shown for other cyanobacterial strains^25,27,43^.

Similar to a previous quantitative study, the majority of identified proteins were related to metabolic processes^30^. Due to optimizations performed in the experimental protocol, the total number of proteins identified in the present study was much higher (1877 vs 546), a 3.5-fold increase compared with our last publication using the FASP + SP3 protocol^30^. These results highly improved the quality of data (e.g., more peptides identified, fewer gaps in the proteomic profile comprising six replicates per condition tested). We were now able to identify several ribosomal proteins and others involved in photosynthesis and respiration, which are essential in primary metabolic functions, as well as proteins associated with phycobilisomes, elongation factors, and transporter proteins.

Previous studies performed on the proteome analysis of the adhesion and biofilm formation of other species also identified the presence of several proteins with disordered domains as DEPs^30,46^. Moreover, one of them is related to protein secretion (A0A522XHL7). PRC domain-containing proteins are involved in photosynthesis activity mainly by transferring electrons within the cyclic electron transport pathway. Produced by all photosynthetic organisms, carotenoids act as light-harvesting pigments and as protectors against oxidative stress^47^. Indeed, the photoactivation of the soluble orange carotenoid protein is the initial step for photoprotective mechanisms, which decrease the excitation energy that arrives at the photochemical centers by increasing thermal dissipation^48^. Cyanophycin is a water-insoluble peptide and a valuable exogenous substrate, which enables cyanobacteria to store nitrogen in environments subjected to fluctuating nitrogen supply^49^. A previous proteomic study performed on *Microcoleus* cyanobacteria also showed cyanophycinase expression throughout biofilm growth, suggesting active utilization of cyanophycin granules to cope with fluctuations in nutrient supply^50^. Glutathione-dependent formaldehyde dehydrogenase is one of the three pathways for the detoxification of formaldehyde widely distributed in biological processes^51^. Formaldehyde can be generated endogenously during redox processes, including during the enzymatic demethylation of methylated nucleic acids and proteins, leading to cellular dysfunction^52^. A study on *Acinetobacter baumannii 8399* showed the induction of *adhC1* gene, which encodes for a glutathione-dependent formaldehyde dehydrogenase, under iron limitation and its repression when the cells are cultured in the presence of free inorganic iron^53^. Moreover, in *Paracoccus denitrificans*, the expression of the gene encoding this enzyme is increased when either formaldehyde or metabolic sources of this compound, such as methanol, are present^54^. Likewise, in *Saccharomyces cerevisiae* the glutathione-dependent formaldehyde dehydrogenase expression level is increased by the presence of methylated compounds like methyl methanesulphonate or formaldehyde^55^. An additional study^56^ demonstrated that a glutathione-dependent detoxification system was required for formaldehyde resistance and optimal survival of *Neisseria meningitidis* in biofilms, suggesting the role of this enzyme in this complex lifestyle.

Cyanobacterial development on glass at 40 s^−1^ promoted the up-regulation of two phage tail proteins, a MoaD/ThiS family protein and an uncharacterized protein with a disordered domain, when compared to 4 s^−1^. Bacteriophage tail core is important for phage adsorption to the bacterial cell wall and host infection^57^. Although cyanophages have been barely investigated, they play an important role in regulating the dynamics of cyanobacterial communities in aquatic environments^58^. Cyanophages may introduce genes involved in photosynthesis, carbon metabolism, and nutrient acquisition into host cells during lysogeny, thereby modifying the metabolism of cyanobacteria and providing a vehicle for horizontal transfer of genetic material and evolution and diversity of marine cyanobacteria^59,60^. Moreover, they also impact globally in the marine biogeochemical cycles^59,61^. Cyanophages can play an essential role in the control of cyanobacterial blooms^61,62^. For example, phage lysis of *Nodularia* leads to the release of nitrogen and stimulates the growth of *Synechococcus*^61^. Therefore, it is noteworthy the identification of two phage tail proteins in this biofilm development condition due to their critical role in marine environments. Receptor-binding proteins could be used in genetic engineering to alter host ranges of cyanophages for biocontrol or to develop bacteriophage-based genetic tools for cyanobacteria^58^. Furthermore, cyanophages could potentially be used to control biofilms, opening up new developments in antifouling solutions. A recent study showed that phage protein reduces biofilm and improves antibiotic action^57^. MoaD and ThiS are sulfur carrier proteins required for the synthesis of cofactors of molybdenum and thiamin. Additionally, sulfur is one of the essential elements which is incorporated in proteins as thiol groups of cysteine residues^63^. Unlike in the previous works^27,30^, the hydrodynamic effect was clearly revealed through PCA analyses, allowing us to consolidate that hydrodynamics may have a more significant effect than surface properties on biofilm formation (Fig. 4). The differential biofilm development observed on glass at 4 s^−1^ and 40 s^−1^ (Fig. 2) can be related to the differential expression of these proteins in this filamentous cyanobacterial strain. Indeed, GO distribution and GO enrichment of DEPs analysis (Fig. 7) revealed two enriched GO terms related to cysteine (cysteine metabolic process and cysteine biosynthetic process) and two additional GO terms related to sulfur metabolic pathways (sulfur compound metabolic process and sulfur amino acid biosynthetic process).

Iron ABC transporter substrate-binding protein, transketolase, transcription termination/antitermination protein NusG, dihydroxy-acid dehydratase, and 30 S ribosomal protein S16 were identified as DEPs on biofilm cells developed on perspex. ABC transporters are vital in the transport of essential substrates across the cytoplasmic membrane^64^. In the case of iron, which is not always readily available from the environment, this metal is a relevant component of key molecules such as cytochromes, and its deficiency can impact ATP production, nucleotide synthesis, and the activity of numerous critical enzymes. Some studies emphasize the role of iron ABC transporters in stress response and virulence^65,66^. In fact, since at 4 s^−1^ the mass transfer of oxygen and nutrients to the inner layers of the biofilm may be reduced, the up-regulation of this protein can be considered as a virulence factor in biofilms. Moreover, a study performed in the anaerobic hyperthermophilic bacterium *Thermotoga maritima* also revealed the up-regulation of an iron ABC transporter on biofilm when compared with planktonic cells^67^. Therefore, they can also be considered as promising targets for antimicrobial strategies. Transketolase is one of the Calvin cycle enzymes involved in biosynthesis, metal ion binding, carbon metabolism, providing a link between glycolysis and the pentose-phosphate pathway^68^. Differential regulation of transketolase has been reported on adhesion, biofilm, and persistence studies. Indeed, a study on *Shewanella oneidensis* showed that transketolase was slightly up-regulated in *luxS* mutant, which developed an undifferentiated, loosely connected biofilm that covered the glass surface more homogenously than the wild type^69^. A proteomic analysis of the biofilm extracellular matrix of *Haemophilus influenzae* biofilms was compared with proteomic analysis of total proteins taken from planktonic bacteria, revealing that transketolase was associated only with the 96 h biofilms^70^. A study performed on *S. aureus* showed that inactivation of a gene that encodes a transketolase leads to a significant decrease in intracellular bacteria proliferation and survival in endothelial cells, associated with a non-persister-like phenotype, suggesting that transketolase is required for bacterial persistence^71^.

All down-regulated proteins found at 4 s^−1^, or up-regulated at 40 s^−1^, on perspex are relevant to critical cyanobacterial processes. Indeed, NusG is an important transcription factor that modulates the initiation, elongation, and termination steps of bacterial transcription by RNA polymerase. Dihydroxy-acid dehydratase is related to amino acid synthesis and acquisition, whereas the ribosomal protein S16 is one of the 21 proteins which belong to the 30 S small subunit of the bacterial ribosome involved in translation and protein synthesis. A study that evaluated the whole-transcriptome shotgun sequencing to compare the *S. pneumoniae* transcriptome in biofilms and dispersed conditions showed down-regulation of dihydroxy-acid dehydratases and 30 S ribosomal protein S16 and up-regulation of ABC transporter proteins under actively dispersed cells^72^. This finding suggests that the behavior of these proteins is susceptible not only to different hydrodynamic conditions of biofilm development but also to different lifestyles (planktonic and biofilm). Interestingly, cyanobacterial dihydroxy-acid dehydratases have also been considered a promising growth inhibition target^73^. GO distribution and enrichment analysis (Fig. 7) also confirmed the involvement of these proteins in essential processes since GO terms as “protein-containing complex subunit organization, macromolecule biosynthetic process, cellular macromolecule biosynthetic process, cellular component organization or biogenesis, cellular component organization, cellular biosynthetic process, biosynthetic process” were identified for this biofilm condition.

Proteomic analyses performed on filamentous cyanobacteria demonstrated different mechanisms for biofilm regulation, which are strongly dependent on cyanobacterial species, as well as on their isolation sites^27,30^. Although ribosomal proteins, proteins involved in photosynthesis and respiration, as well as proteins associated with phycobilisomes, elongation factors, and proteins with transporter activity were found in all these studies, a broad diversity in the DEP’s profile of each strain was observed. Therefore, future studies should focus on different cyanobacterial strains to determine a proteomic profile according to the isolation site and/or the cyanobacterial genus.

The effective management of cyanobacterial growth is vital to restore ecosystem function, as well as to mitigate the biofouling effects. This work demonstrates how hydrodynamic conditions and surface properties affect biofilm development and the protein expression on biofilms cells. Macroscopic biofilm analysis revealed a higher biofilm development at the lowest shear rate, while surface effects were less pronounced. The quantitative proteomic analysis also revealed higher variations between biofilm formed at different shear rates when compared with protein profiles obtained from distinct surfaces. Among all DEPs found between 4 s^−1^ and 40 s^−1^, up-regulated proteins associated with the photosynthesis process and related pathways were identified on both surfaces. However, it is important to highlight the influence of the glutathione-dependent formaldehyde dehydrogenase found on glass. While down-regulated proteins related to essential processes such as transcription and translation were found at 4 s^−1^ on perspex, on glass, these down-regulated proteins were linked to metabolic processes. Interestingly, phage tail proteins, which presented a significant effect on cyanobacteria blooms and aquatic environments, were also identified on glass.

The possible proteomic functions reported in this study were verified by their quantitative and relative expression in all biofilm conditions and supported by statistical analysis. Considering the lack of biomarkers identified in previous works using a similar approach, the role of the different DEPs can only be discussed by their biological signatures, such as protein domains, annotations, and their relationship in enriched pathways.

Further studies should include gene silencing or knockout strains to establish a stronger link between differential expression levels of a given protein and the impact on biofilm development and behavior. However, the methodologies required for performing this work in this type of organisms are still under development. Nevertheless, and even considering the low availability of data, this study demonstrates with statistical significance that some proteins are differentially expressed under the different conditions and these experimental variations induce changes in biofilm development. Although this is a limitation of our work, to the best of our knowledge, it reflects the state of the art of the research in this field. Additionally, two relevant and clear conclusions were achieved. Regarding the proteomic protocol improvement, the sample preparation, including ultrasounds treatment of the biomass in SDT buffer for cell rupture and protein extraction, followed by a combination of FASP and SP3 protocol, notably improves the peptide and protein identification by shotgun proteomics. Moreover, related to the SP3, in this study we demonstrated its suitability to capture and retain a higher number of peptides originating from complex samples. Regarding the effect of surface properties and hydrodynamic conditions on cyanobacterial biofilm development, it was shown that hydrodynamics had a stronger effect causing the up-regulation of 13 proteins and the downregulation of 7 proteins when biofilms developed at a shear rate value of 4 s^−1^ with 40 s^−1^ are compared (Fig. 6).

Moreover, the isolation of marine organisms (namely marine bacteria) from fouled submerged surfaces (for instance ship hulls and pontoons) and the determination of their proteomic profile can provide information about the metabolic pathways that are activated during biofilm formation under these conditions. Since it is known that cyanobacteria interfere with other organisms in their communities through the release of compounds into the surrounding medium^31^, the identification and in vitro production of these compounds can be exploited towards the development of marine antifouling strategies where the growth of different fouling organisms can be inhibited^32,74–76^. This concept has been applied in the use of nostocarboline, a carboline alkaloid from *Nostoc* 78-12 A, as an environmentally friendly candidate for antifouling coating against both prokaryotic and eukaryotic photosynthetic organisms^77,78^. Additionally, other metabolites derived from cyanobacteria have been explored as new candidates for antifouling coatings, such as the diterpenoid comnostin B, isolated from *Nostoc commune*, which shown a molluscicidal effect^79^, or the *epi*-hapalindole E from *Fischerella* sp., which exhibited antibacterial, antifungal, antialgal and activities against mammalian cells^80^. Previous studies with portoamides, cyclic dodecapeptides isolated from the cyanobacterium *Phormidium* sp. LEGE 05292, also reveal a broad-spectrum bioactivity towards diverse biofouling species, given their allelopathic effect on the microalga *Chlorella vulgaris*^31^ and the high effectiveness in the prevention of the attachment of mussel larvae^32^, showing potential to be incorporated as an active ingredient in antifouling coatings.

This study is particularly important given the reduced number of studies addressing filamentous cyanobacterial biofilm development in conditions that mimic the natural environment and that are able to integrate macroscopic biofilm techniques and proteomic approaches. The relatively high number of uncharacterized proteins that were identified in this study is further proof that this area requires further interdisciplinary work so that new insights on biofilm formation and mitigation in marine environments can be obtained.

## Methods

### DNA extraction, Amplification (PCR) and sequencing

A 50 ml culture of a cyanobacterium previously classified as unidentified filamentous Synechococcales LEGE 06021^39^ was grown and cells were harvested after 2 - 3 weeks. Total genomic DNA was extracted using the commercial PureLink Genomic DNA Mini Kit (Invitrogen, USA), according to the manufacturer’s instructions provided for Gram-negative bacteria. To obtain the sequence of 16 S rRNA gene, PCR amplification was performed using the 27F^81^, CYA359F, CYA781R^82^ and 1494R^81^ oligonucleotide primers. PCR reactions were performed in a final volume of 20 µl containing 1x Green GoTaq Flexi Buffer, 2.5 mM of MgCl_2_, 125.0 mM of each deoxynucleotide triphosphate, 1.0 µM of each primer, 0.5 U of GoTaq Flexi DNA Polymerase (Promega, Madison, WI, USA), 10 mg.ml^−1^ of bovine serum albumin (BSA), and 10–30 ng of template DNA, on a Veriti™ 96-Well Thermal Cycler. The PCR conditions were as follows: initial denaturation at 95 °C for 2 min, followed by 35 cycles of denaturation at 95 °C for 1 min, annealing at 50 °C for 45 s, and extension at 72 °C for 1 min, with a final extension step at 72 °C for 5 min. The PCR reactions were performed in duplicate. PCR products were separated by 1.5% agarose gel stained with SYBR^®^ safe (Invitrogen, Waltham, MA, USA) and DNA fragments with the expected size were excised and purified using NZYGelpure (NzyTech, Genes and Enzymes, Lisbon, Portugal) according to the manufacturer’s instructions. Sequencing was performed at GATC Biotech (Ebersberg, Germany) and the nucleotide sequence obtained was manually inspected for quality and assembled using the Geneious 11.1.5 software (Biomatters Ltd., Auckland, New Zealand). Possible chimera formation during the sequences was checked using the software DECIPHER^83^. The sequence obtained was inserted in the BLASTn (Basic Local Alignment and Search Tool for Nucleotides) database and the results were analysed. The sequence associated with this study was deposited in the GenBank database under the accession number ON258650.

### Phylogenetic analysis

A total of 61 sequences were used in the final analysis, including 2 strains of *Gloeobacter violaceus* as outgroup, 58 sequences of cyanobacteria, including type, reference and related strains belonging to the orders Pseudanabaenales and Synechococcales were retrieved from GenBank (National Center for Biotechnology Information, NCBI, Bethesda, MD, USA), and 1 sequence of unidentified filamentous Synechococcales LEGE 06021 obtained in this work. Multiple sequence alignment was carried out using ClustalW in MEGA7^84,85^, and sequences were manually proofread and edited. The best fit model was assessed using jModelTest 2.1.10^86^ according to the Bayesian information criterion (BIC) and Akaike information criterion (AIC) scores. Maximum likelihood (ML) analysis was carried out using substitution model GTR + G + I with 1000 bootstrap resampling replicates using the MEGA7 software^85^. The final phylogenetic tree was visualized and edited on iTOL (Interactive Tree of Life)^87^.

### Organism and inoculum preparation

The cyanobacterium previously identified as unidentified filamentous Synechococcales LEGE 06021 was obtained from the Blue Biotechnology and Ecotoxicology Culture Collection (LEGE-CC) located at CIIMAR, Portugal^39^. This cyanobacterial strain was firstly isolated from a sample scraped from a wave-exposed rock in the intertidal zone at Coxos beach, Santo Isidoro, Portugal (39.00455 N 9.425842 W). Cells were grown in 750 ml culture in Z8 medium^88^ supplemented with 25 g.l^−1^ of synthetic sea salts (Tropic Marin) and B_12_ vitamin (Sigma Aldrich, Merck, Saint Louis, MO, USA). Cultures were performed under 14 h light (10–30 μmol photons m^−2^ s^−1^)/10 h dark cycles, at 25 °C.

### Biofilm formation

In order to assess the biofouling potential on different surfaces, as well as to evaluate their protein expression profiles, biofilms were developed on glass (Vidraria Lousada, Lda, Portugal) and perspex (Neves & Neves, Lda, Portugal) coupons (1 cm^2^). These represent typically submerged artificial surfaces found on different apparatus in aquatic environments such as aquaculture equipment, measuring devices and sensors, flotation spheres, moored buoys, underwater cameras, or even in underwater windows of boats^8,89,90^ that can be affected by biofouling. Moreover, a previous study shows that these surfaces present different properties since perspex is relatively hydrophobic compared to glass^43^. Surface sterilization and preparation were performed as follows. Glass and perspex coupons were immersed in a solution of 2% (vol/vol) TEGO 2000® industrial detergent (Johnson Diversey, Northampton, United Kingdom)^91^ and sterile distilled water for 20 min under agitation (150 rpm). In order to remove any remaining disinfectant, coupons were rinsed in sterile distilled water and air-dried. Additionally, glass coupons were autoclaved (121 °C, 15 min)^92^. After drying, all coupons were aseptically pre-weighted. Biofilm formation was evaluated on agitated 12-well microtiter plates (VWR International, Carnaxide, Portugal), since this platform was shown to mimic the hydrodynamic conditions found in marine environments^43^. Transparent double-sided adhesive tape was used to fix the coupons, and once the tape was placed in the wells, all coupons plates were subjected to UV sterilization for 30 min after which the sterile coupons were fixed. Moreover, to assess the hydrodynamic effect on protein expression, cyanobacterial biofilm formation at different shear rates was also tested. Briefly, to attain the shear rate values that can mimic aquatic environments, such as those found in a ship hull in a harbor and in partially submerged or even moored equipment and devices, microtiter plates were incubated at 25 °C in an orbital shaker with a 25 mm orbital diameter (Agitorb 200ICP, Norconcessus, Portugal) at 185 rpm (achieving the average shear rate of 40 s^−1^)^43^. Biofilm development was also assessed at lower shear rate conditions (average shear rate of 4 s^−1^, achieved at 40 rpm) since lower fluid velocities can promote marine biofouling^93,94^. As chlorophyll *a* concentration is commonly used as biomass indicator in aquatic environments, the cyanobacterial suspension was adjusted to a chlorophyll *a* concentration of 1.5 µg.ml^−1^ to inoculation. Briefly, 2 ml of cyanobacterial suspension were incubated at 4 °C in the dark for a maximal chlorophyll *a* extraction. After 24 h, the samples were centrifuged at 3202 g for 5 min at room temperature, and the supernatant was transferred to a glass cuvette. The absorbance at 750 nm (turbidity), 665 nm (chlorophyll *a*), and 652 nm (chlorophyll *b*) was determined using a V-1200 spectrophotometer (VWR International China Co., Ltd, Shanghai, China). The chlorophyll *a* concentration was calculated through the following equation^95^:

1 $${{{\mathrm{Chl}}}}\;a\left( {\mu {{{\mathrm{g}}}}.{{{\mathrm{ml}}}}^{ - 1}} \right) = 16.29 \times {{{\mathrm{A}}}}^{665}-8.54 \times {{{\mathrm{A}}}}^{652}$$

These measurements were assessed in triplicate, and dilutions were performed using Z8 medium supplemented with 25 g.l^−1^ of synthetic sea salts and vitamin B_12_. Thus, a volume of 3 ml of adjusted cyanobacterial suspension was inoculated in each well, in which coupons of each surface were previously fixed with transparent double-sided adhesive tape. Biofilm development was followed for 49 days (seven weeks) since it is accepted that a two-month interval for maintenance is the minimum duration for economically viable underwater monitoring systems^43,90^. During this incubation time, the medium was replaced twice a week. Moreover, to mimic real light exposure periods, a photoperiod of 14 h light (8–10 µmol photons m^−2^ s^−1^)/10 h dark cycles) was applied.

### Biofilm analysis

Every seven days, biofilm analysis was performed and three coupons for each surface and hydrodynamic condition were analysed. The culture medium was carefully removed, and the wells were filled with 3 ml of sterile sodium chloride solution (8.5 g.l^−1^)^43^. The solution was carefully removed to eliminate loosely attached cyanobacteria and subsequently, the wells were filled again with 3 ml of sterile sodium chloride solution to evaluate the cyanobacterial biofilms thickness and structure by OCT. In order to complement the characterization of cyanobacterial biofilms, the determination of their wet weight and chlorophyll *a* content was also performed.

#### Optical coherence tomography

Images from cyanobacterial biofilms developed on both surfaces and under the different hydrodynamic conditions were captured. For each coupon, 2D imaging was performed with a minimum of 3 fields of view to ensure the accuracy and reliability of the results obtained^43^. Image analysis was performed using a routine developed in the Image Processing Toolbox from MATLAB 8.0 and Statistics Toolbox 8.1 (The MathWorks, Inc., Natick, Massachusetts, USA)^27^. The mean of biofilm thickness was calculated based on the distance between the biofilm bottom and the upper contour line according to the following Eq. (2):

2 $$\bar L_F = \frac{1}{N}\mathop {\sum}\limits_{i = 1}^N {L_{F,i}}$$

where $L_{F,i}$ is a local biofilm thickness measurement at location *i, N* equals the number of thickness measurements, and $\bar L_F$ is the mean biofilm thickness.

#### Wet weight determination and chlorophyll a quantification

To determine the wet weight, coupons were detached from the wells, weighed, and the biofilm wet weight was obtained as the difference from the initial coupon weight (determined prior to inoculation)^30^. Subsequently, cyanobacterial cells were detached from the coupons by immersing each coupon in 2 ml of 8.5 g.l^−1^ sodium chloride solution and vortexing. The suspensions were incubated for 24 h at 4 °C in the dark, and chlorophyll *a* determination was performed as previously described through Eq. 1.

#### Statistical analysis of biofilm development

A total of six replicates (two biological assays with three technical replicates each) were analysed. Data analysis was performed using the statistical program GraphPad Prism® for Windows, version 6.01 (GraphPad Software, Inc., San Diego, CA, USA), and results were compared using unpaired t-tests with a confidence level of 95% (*; *P* < 0.05).

### Proteomics analysis

#### Protein extraction and sample preparation for proteomic analysis

At the last sampling time (49 days), biofilm samples of unidentified filamentous Synechococcales LEGE 06021 were recovered for proteomic analysis^30^. Cyanobacterial biofilms were detached from the coupons by immersing each coupon in 2 ml of 8.5 g.l^−1^ sodium chloride solution and vortexing. The pooled samples from four coupons of the same condition were centrifuged at 3202 g for 10 min at room temperature, and the pellet of biomass from 24 biofilm samples comprising six independent replicates from four different growing conditions were kept at −80 °C for further processing. The amount of sample was calculated, and an appropriate volume of SDT buffer (0.5 g FW/ml SDT) + Protease inhibitors (PIs, Roche, 11697498001, Basel, Switzerland) was added to the samples, sonicated 10 × 3 s and 23 kHz – 105 µm (amplitude) and incubated for 4 h at room temperature. Afterward, samples were heated for 3 min at 95 °C and subsequently centrifuged at 16,000 g for 20 min. Finally, the supernatant was collected, and total protein concentration was measured indirectly by optical density (OD) at 280 nm using a DeNovix DS-11 Spectrophotometer (DeNovix Technologies, Wilmington, Delaware, USA). Samples containing the extracted protein were stored for 24 h at −20 °C.

The extracted proteins were processed according to Romeu et al.^30^, which used a modified version of two distinct protocols based on FASP protocol described by^96^ and the SP3 technology^97^. Herein, the samples containing 40 µg of the extracted proteins were processed by the FASP using centrifugal filter units with nominal molecular weight limit (NMWL) of 30 kDa (MRCPRT030, Millipore, Billerica, MA, USA), whereas 200 µg of the extracted proteins were reduced prior the digestion with trypsin in the SP3 protocol^30^.

#### LC-MS/MS analysis

Considering both sample preparation methods are complementary, tryptic peptides from both methods were mixture in the same tube to a final concentration of 0.1 µg and 0.5 µg from FASP and SP3, respectively. Then, protein digests from both protocols were analysed in the same run with a nano LC-MS/MS, composed by an Ultimate 3000 liquid chromatography system coupled to a Q-Exactive Hybrid Quadrupole – Orbitrap mass spectrometer (Thermo Scientific, Bremen, Germany)^98^. Separation was performed in a 15 cm by 75 μm inner diameter EASY-Spray column (ES800, PepMap RSLC, C18, 3 μm, Thermo Scientific, Bremen, Germany) at 300 nl min^−1^ by generated by mixing A: 0.1% FA and B: 80% ACN, with the following gradient: 5 min (2.5% B to 10% B), 60 min (10% B to 35% B), 5 min (35% B to 99% B) and 5 min (hold 99% B). Subsequently, the column was equilibrated with 2.5% B for 12 min. Data acquisition was controlled by Xcalibur 4.0 and Tune 2.8 software (Thermo Scientific, Bremen, Germany). The specific LC-MS parameters were full scan settings: 70k resolution (m/z 200), AGC target 3e6, maximum injection time 50 ms. dd settings: minimum AGC target 8e3, intensity threshold 7.3e4, charge exclusion: unassigned, 1, 8, >8, peptide match preferred, exclude isotopes on, dynamic exclusion 20 s. MS2 settings: microscans 1, resolution 35k (m/z 200), AGC target 2e5, maximum injection time 110 ms, isolation window 2.0 m/z, isolation offset 0.0 m/z, spectrum data type profile.

The mass spectrometry proteomics data have been deposited to the ProteomeXchange Consortium via the PRIDE^99^ partner repository with the data set identifier PXD029048.

#### Protein identification

The raw data were analysed and processed using the Proteome Discoverer 2.2.0.388 software (Thermo Scientific) and searched against the UniProt database for Cyanobacteria taxonomic selection (2018_07 release). The Sequest HT search engine was used for protein identification. The ion mass tolerance was 10 ppm for precursor ions and 0.02 Da for-fragment ions. Maximum allowed missing cleavage sites was set to 2. Cysteine carbamidomethylation was defined as a constant modification. Methionine oxidation and protein N-terminus acetylation were defined as variable modifications. Peptide confidence was set to high. The processing node Percolator was enabled with the following settings: maximum delta Cn 0.05; decoy database search target false discovery rates (FDR) 1%, validation was based on q-value. Protein label-free quantitation was performed with the Minora feature detector node at the processing step. Precursor ions quantification was performed at the processing step with the following parameters: unique plus razor peptides were considered for quantification, and precursor abundance was based on intensity.

#### Differential protein expression and enrichment

Statistical analyses applied to the proteomic data were performed using R software version 4.0.0.^100^. The Differentially Expressed Proteins (DEPs) and/or gene enrichment was assessed using the “DEP” R package^101^, based on the *limma* algorithm^102^. Similar to previous work^30^, the missing values were filtered according to the fraction of protein found in each paired comparison with a cut-off of 0.33%, considering the possibility to have a minimum of 4 out of 6 replicates at least one condition. Imputation of missing values was performed considering a non-random distribution (MNAR) using “MinProb” as an imputation function. Significant differences between conditions for each condition were paired tested by protein-wise linear models and empirical Bayes statistics. Finally, adjusted *P* value (alpha = 0.05) and the log2 fold change (lfc = log2(1.5), log2 fold change >0.6) were used as a filter threshold to classify them as DEPs or enriched genes.

Functional classification (gene ontology) was carried out using the OmicsBox software version 1.4.11 (https://www.biobam.com/omicsbox/). Additional functional analyses to determine over/under-expressed pathways were performed to the categorical GO terms corresponding to DEPs, using the hypergeometric distribution significance test (Fisher’s exact test, *P* < 0.05).

## Supplementary information

Supplementary Material

Supplementary Table 1

Supplementary Table 2

## Data Availability

The sequence associated with the cyanobacterial strain used in this study was deposited in the GenBank database under the accession number ON258650. The mass spectrometry proteomics data have been deposited to the ProteomeXchange Consortium via the PRIDE partner repository with the data set identifier PXD029048. All other data that support the findings of this study are available from the corresponding author upon reasonable request.
