# Supplementary material for: Hydrodynamic conditions affect the proteomic profile of marine biofilms formed by filamentous cyanobacterium
Source: NPJ Biofilms Microbiomes. 2022 Oct 17;8:80. doi: 10.1038/s41522-022-00340-w (PMC9576798; doi:10.1038/s41522-022-00340-w)
Supplement: Supplementary file 1 — Supplementary Material [file 41522_2022_340_MOESM1_ESM.pdf]

ADDITIONAL INFORMATION

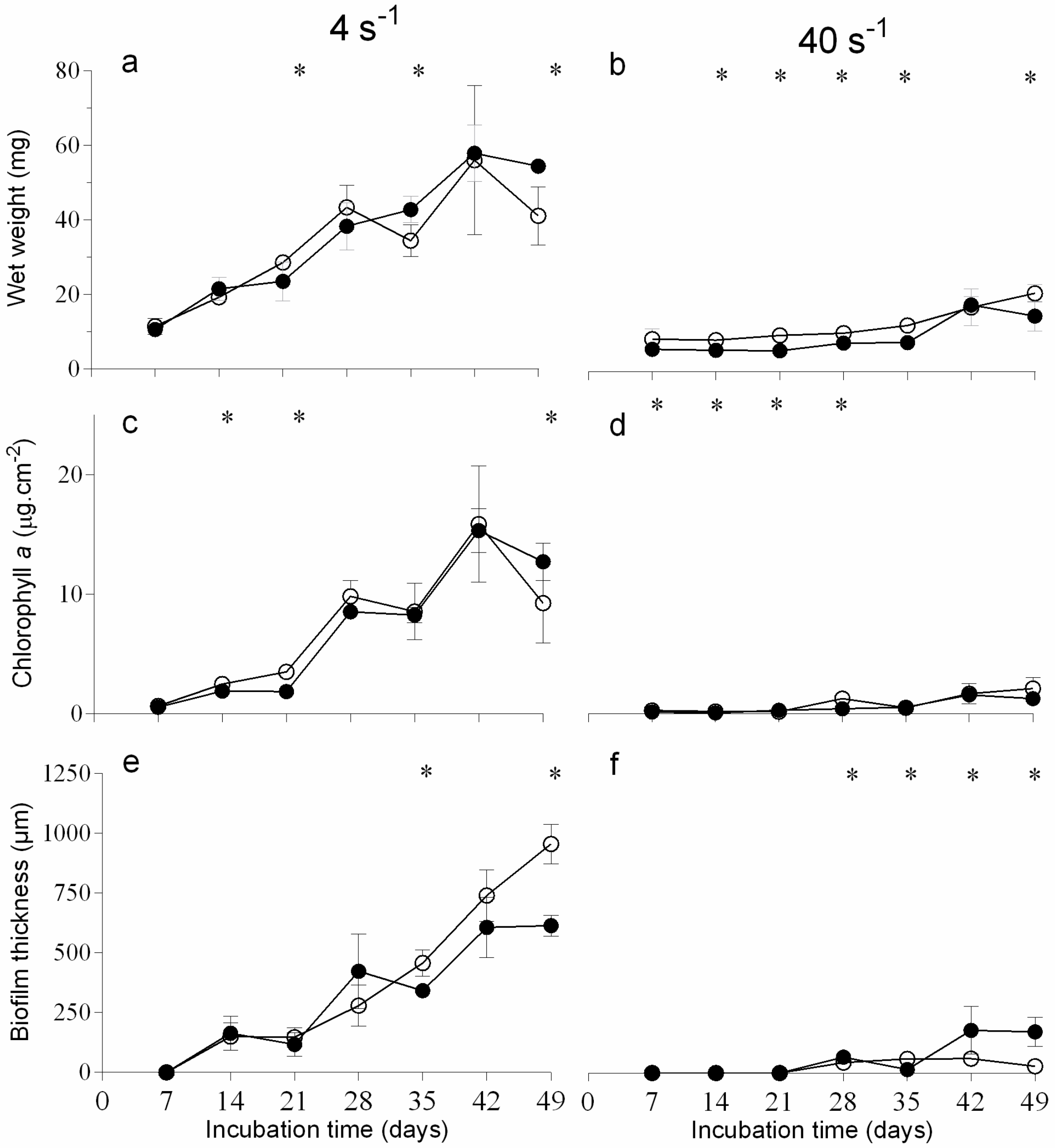

Supplementary Figure 1. Evaluation of unidentified filamentous Synechococcales LEGE 06021 biofilm development under the same hydrodynamic condition on different surfaces. The parameters analysed refer to wet weight (a, b), chlorophyll *a* quantification (c, d) and biofilm thickness (e, f). Biofilms were formed at 4 s^{-1} (a, c, e) or 40 s^{-1} (b, d, f) on two different surfaces, glass (closed circles) and perspex (open circles) for 49 days. Standard deviations from two biological assays with three replicates each are represented. Symbol * indicates statistically different values for $P < 0.05$ (unpaired t-tests) at each incubation time.

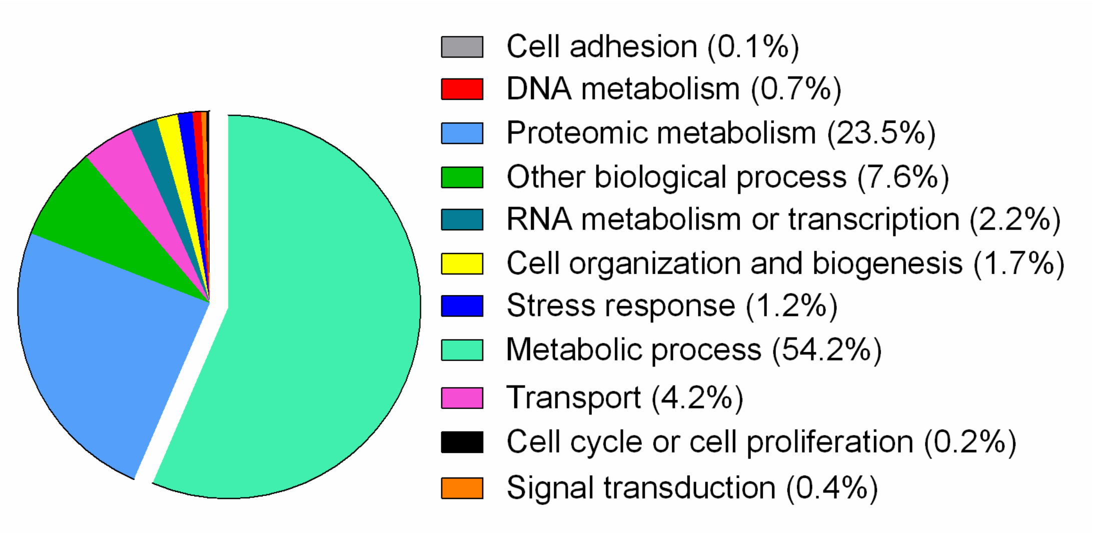

Supplementary Figure 2. Distribution of total proteins identified according to the biological process (34.8 % of proteins identified without an associated biological process are not shown in this graph).

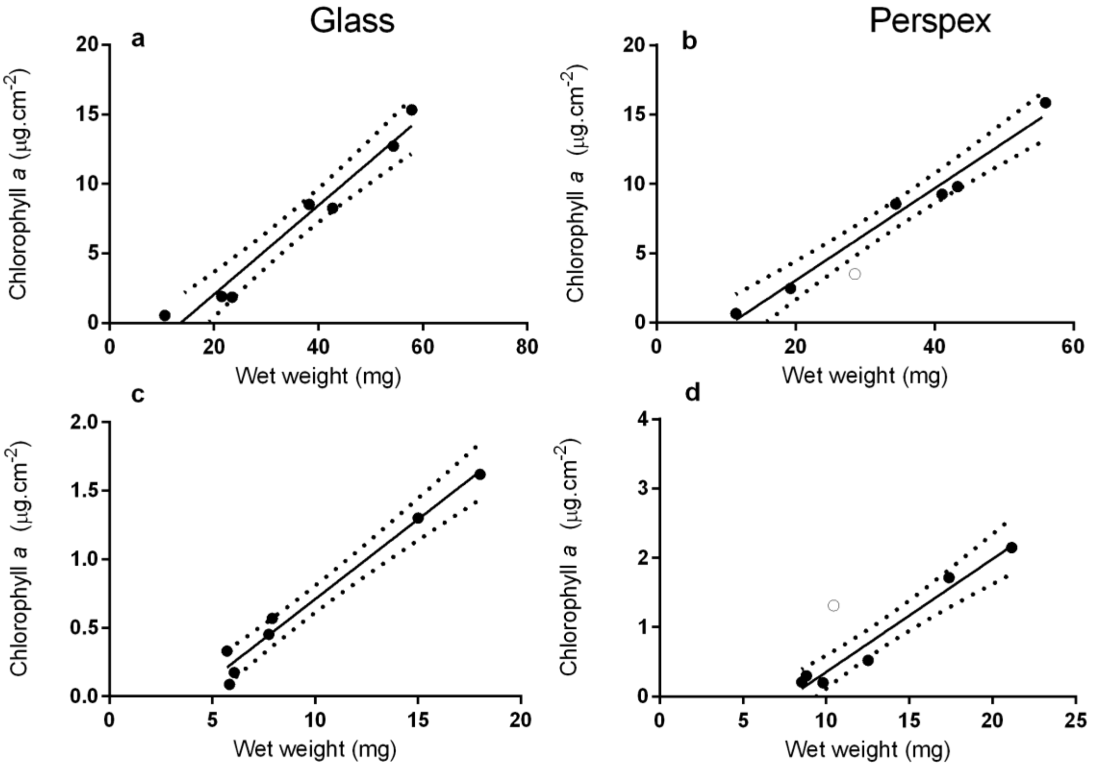

Supplementary Figure 3. Correlation between wet weight and chlorophyll *a* content on biofilm development from unidentified filamentous Synechococcales LEGE 06021. Biofilm development in glass (a, c) and perspex (b, d) at two different shear rates, 4 s^{-1} (a, b) and 40 s^{-1} (c, d). The full line represents the linear regression, and the dashed line represents the confidence limits (95 %). Closed symbols refer to data points that were considered for the regression (all except two), and open symbols refer to data points that were excluded from the regression.
